# Supplementary material for: Molecular Epidemiology of Pasteurella multocida Associated with Bovine Respiratory Disease Outbreaks
Source: Animals (Basel). 2022 Dec 24;13(1):75. doi: 10.3390/ani13010075 (PMC9817976; doi:10.3390/ani13010075)
Supplement: Supplementary file 1 [file animals-13-00075-s001.zip › animals-2074248-supplementary.pdf]

Table S1. Details of the 125 BRD outbreaks and the 170 *P. multocida* isolates analyzed.

Outbreak	Province	Farm	Date	No. of isolates	Cap:LPS:VT	ST* RIRDC
**1**	A Coruña	Granja 2	03/01/2020	1	A:L3:VT1	ST79
**2**	Ávila	Granja 86	26/11/2020	1	A:L3:VT2	
**3**	Ávila	Granja 86	17/06/2021	1	A:L3:VT2	
**4**	Ávila	Granja 205	25/10/2021	4	A:L3:VT1	ST13
**5**	Ávila	Granja 214	17/11/2021	1	A:L3:VT2	ST79
**6**	Barcelona	Granja 201	06/10/2021	1	A:L3:VT1	ST13
**7**	Barcelona	Granja 220	17/12/2021	1	A:L3:VT1	
**8**	Burgos	Granja 67	01/09/2020	1	B:L2:VT5	ST322
**9**	Burgos	Granja 152	22/04/2021	1	A:L3:VT1	ST13
**10**	Burgos	Granja 225	04/01/2022	1	A:L3:VT1	
**11**	Cáceres	Granja 88	27/11/2020	1	A:L3:VT1	ST13
**12**	Cáceres	Granja 89	27/11/2020	4	A:L3:VT1	
**13**	Cáceres	Granja 187	24/08/2021	1	A:L3:VT2	ST79
**14**	Castellon	Granja 227	11/01/2022	1	A:L3:VT1	ST13
**15**	Gipuzkoa	Granja 173	23/06/2021	1	A:L3:VT1	ST79
**16**	Girona	Granja 34	12/05/2020	1	A:L3:VT1	ST13
**17**	Girona	Granja 47	05/06/2020	1	A:L3:VT1	
**18**	Girona	Granja 55	24/07/2020	1	A:L3:VT1	ST79
**19**	Girona	Granja 80	17/11/2020	1	A:L3:VT1	
**20**	Girona	Granja 34	28/01/2021	1	A:L3:VT1	
**21**	Girona	Granja 212	10/11/2021	1	A:L3:VT7	ST13
**22**	Guadalajara	Granja 1	03/01/2020	1	A:L3:VT1	ST79
**23**	Guadalajara	Granja 222	22/12/2021	1	A:L3:VT1	
**24**	Huesca	Granja 33	08/05/2020	1	A:L3:VT1	
**25**	Huesca	Granja 66	28/08/2020	1	A:L3:VT1	
**26**	Huesca	Granja 113	10/02/2021	1	A:L3:VT2	ST79
**27**	Huesca	Granja 114	10/02/2021	1	A:L3:VT2	
**28**	Huesca	Granja 144	06/04/2021	1	A:L3:VT1	
**29**	Huesca	Granja 160	20/05/2021	1	A:L3:VT1	ST13
**30**	Huesca	Granja 193	13/09/2021	4	A:L3:VT1	
**31**	Huesca	Granja 229	19/01/2022	1	A:L3:VT1	
**32**	Huesca	Granja 230	01/02/2022	1	A:L3:VT1	
**33**	La Rioja	Granja 30	30/04/2020	4	A:L3:VT1	ST79
					A:L3:VT2	ST79
					A:L3:VT4	ST79
**34**	La Rioja	Granja 8	30/04/2020	1	A:L3:VT1	ST79
**35**	La Rioja	Granja 30	19/05/2020	1	A:L3:VT4	
**36**	La Rioja	Granja 195	28/09/2021	1	A:L3:VT1	

**37**	Las Palmas	Granja 45	04/06/2020	1	A:L3:VT1	ST13
**38**	Las Palmas	Granja 181	10/08/2021	1	A:L3:VT1	ST79
**39**	Las Palmas	Granja 181	18/08/2021	4	A:L3:VT1	
**40**	Las Palmas	Granja 224	30/12/2021	1	A:L3:VT2	ST79
**41**	León	Granja 21	03/03/2020	1	A:L3:VT1	ST79
**42**	León	Granja 153	29/04/2021	1	A:L3:VT1	
**43**	Lleida	Granja 46	04/06/2020	1	A:L3:VT1	
**44**	Lleida	Granja 96	22/12/2020	4	A:L3:VT1 (4)	ST79
**45**	Lleida	Granja 99	31/12/2020	1	A:L3:VT1	
**46**	Lleida	Granja 216	02/12/2021	1	A:L3:VT2	ST79
**47**	Lleida	Granja 217	03/12/2021	1	A:L3:VT2	
**48**	Lleida	Granja 228	14/01/2022	1	A:L3:VT2	
**49**	Lleida	Granja 231	01/02/2022	1	A:L3:VT1	
**50**	Lugo	Granja 23	04/03/2020	1	A:L3:VT1	ST79
**51**	Murcia	Granja 98	22/12/2020	4	A:L3:VT1	ST79
**52**	Murcia	Granja 119	18/02/2021	1	A:L3:VT1	
**53**	Murcia	Granja 161	25/05/2021	4	A:L3:VT1	
**54**	Murcia	Granja 219	16/12/2021	1	A:L3:VT1	
**55**	Murcia	Granja 221	17/12/2021	1	A:L3:VT1	
**56**	Navarra	Granja 6	15/01/2020	1	A:L3:VT1	
**57**	Navarra	Granja 9	22/01/2020	1	A:L3:VT1	
**58**	Navarra	Granja 10	24/01/2020	1	A:L3:VT1	
**59**	Navarra	Granja 6	25/11/2020	1	A:L3:VT1	
**60**	Navarra	Granja 94	17/12/2020	1	A:L3:VT1	
**61**	Navarra	Granja 94	19/01/2021	1	A:L3:VT1	
**62**	Navarra	Granja 127	03/03/2021	1	A:L3:VT1	ST79
**63**	Navarra	Granja 164	04/06/2021	2	A:L3:VT1	
**64**	Navarra	Granja 167	10/06/2021	1	A:L3:VT2	ST79
**65**	Navarra	Granja 172	23/06/2021	1	A:L3:VT1	
**66**	Navarra	Granja 174	23/06/2021	3	A:L3:VT1	
**67**	Navarra	Granja 94	04/08/2021	1	A:L3:VT1	
**68**	Navarra	Granja 180	06/08/2021	1	A:L3:VT1	
**69**	Navarra	Granja 94	27/08/2021	1	A:L3:VT1	
**70**	Navarra	Granja 213	10/11/2021	1	A:L3:VT1	
**71**	Navarra	Granja 232	02/02/2022	1	A:L3:VT1	
**72**	Palencia	Granja 191	09/09/2021	1	A:L3:VT1	ST79
**73**	Salamanca	Granja 19	27/02/2020	1	A:L3:VT2	ST79
**74**	Salamanca	Granja 57	10/08/2020	1	A:L3:VT1	ST79
**75**	Salamanca	Granja 81	17/11/2020	1	F:L3:VT6	ST9
**76**	Salamanca	Granja 87	26/11/2020	1	A:L3:VT2	
**77**	Salamanca	Granja 163	27/05/2021	1	A:L3:VT1	
**78**	Salamanca	Granja 19	17/06/2021	1	A:L3:VT2	
**79**	Segovia	Granja 20	27/02/2020	1	A:L3:VT1	ST13
**80**	Segovia	Granja 27	13/03/2020	1	A:L3:VT1	

**81**	Segovia	Granja 95	18/12/2020	1	A:L3:VT1	
**82**	Segovia	Granja 203	15/10/2021	1	A:L6:VT3	ST206
**83**	Segovia	Granja 218	16/12/2021	1	A:L3:VT1	
**84**	Soria	Granja 18	25/02/2020	1	A:L3:VT1	ST13
**85**	Soria	Granja 166	09/06/2021	4	A:L3:VT1	
**86**	Soria	Granja 215	24/11/2021	1	A:L3:VT1	
**87**	Teruel	Granja 154	29/04/2021	4	A:L3:VT1 (3);	ST13
					A:L3:VT2 (1)	ST79
**88**	Teruel	Granja 226	07/01/2022	1	A:L3:VT1	
**89**	Toledo	Granja 38	20/05/2020	4	A:L3:VT1	ST79
**90**	Toledo	Granja 41	20/05/2020	1	A:L6:VT3	ST206
**91**	Toledo	Granja 40	20/05/2020	4	A:L3:VT1	
**92**	Toledo	Granja 82	24/11/2020	1	A:L3:VT2	ST79
**93**	Toledo	Granja 91	04/12/2020	1	A:L3:VT1	
**94**	Toledo	Granja 92	04/12/2020	1	A:L3:VT1	
**95**	Toledo	Granja 116	16/02/2021	4	A:L3:VT1	
**96**	Toledo	Granja 82	04/03/2021	1	A:L3:VT1	
**97**	Toledo	Granja 82	12/08/2021	1	A:L3:VT2	
**98**	Toledo	Granja 223	29/12/2021	1	A:L3:VT1	
**99**	Toledo	Granja 233	03/02/2022	1	A:L3:VT2	
**100**	Valencia	Granja 190	31/08/2021	1	A:L3:VT1	
**101**	Valencia	Granja 200	05/10/2021	1	A:L3:VT1	ST79
**102**	Valencia	Granja 211	05/11/2021	1	A:L3:VT2	ST79
**103**	Valladolid	Granja 22	04/03/2020	1	A:L3:VT1	
**104**	Valladolid	Granja 14	02/12/2020	1	A:L3:VT1	ST13
**105**	Valladolid	Granja 14	11/03/2021	1	A:L3:VT1	
**106**	Valladolid	Granja 147	08/04/2021	1	A:L3:VT1	
**107**	Valladolid	Granja 93	21/04/2021	1	A:L3:VT1	
**108**	Valladolid	Granja 147	14/05/2021	1	A:L3:VT1	
**109**	Valladolid	Granja 171	17/06/2021	1	A:L3:VT1	
**110**	Zamora	Granja 5	10/01/2020	1	A:L3:VT1	
**111**	Zamora	Granja 5	29/01/2020	1	A:L3:VT1	
**112**	Zamora	Granja 43	22/05/2020	4	A:L3:VT1	ST79
**113**	Zamora	Granja 49	12/06/2020	1	A:L3:VT1	
**114**	Zamora	Granja 50	17/06/2020	1	A:L3:VT2	ST79
**115**	Zamora	Granja 101	18/01/2021	1	A:L3:VT1	
**116**	Zamora	Granja 132	11/03/2021	1	A:L3:VT1	
**117**	Zamora	Granja 142	26/03/2021	1	A:L3:VT1	
**118**	Zamora	Granja 5	20/05/2021	1	A:L3:VT1	
**119**	Zamora	Granja 176	08/07/2021	1	A:L3:VT2	
**120**	Zamora	Granja 234	04/02/2022	1	A:L3:VT1	
**121**	Zaragoza	Granja 11	28/01/2020	1	A:L3:VT1	
**122**	Zaragoza	Granja 65	27/08/2020	1	A:L3:VT1	ST13
**123**	Zaragoza	Granja 65	10/11/2020	1	A:L3:VT1	

**124**	Zaragoza	Granja 149	14/04/2021	1	A:L3:VT1
**125**	Zaragoza	Granja 150	16/04/2021	1	A:L3:VT1

*One isolate representative of the different genotypes (capsular-LPS and virulotypes) identified in each province was analyzed
